# Thermodynamic theory of the plasmoelectric effect

**DOI:** 10.1038/srep23283

**Published:** 2016-03-18

**Authors:** Jorik van de Groep, Matthew T. Sheldon, Harry A. Atwater, Albert Polman

**Affiliations:** 1 Center for Nanophotonics, FOM Institute AMOLF, Science Park 104, 1098 XG Amsterdam, Netherlands; 2 Department of Chemistry, Texas A&M University, College Station, TX 77843, USA; 3 Thomas J. Watson Laboratories of Applied Physics, California Institute of Technology, MC 128–95, Pasadena, CA 91125, USA

## Abstract

Resonant metal nanostructures exhibit an optically induced electrostatic potential when illuminated with monochromatic light under off-resonant conditions. This plasmoelectric effect is thermodynamically driven by the increase in entropy that occurs when the plasmonic structure aligns its resonant absorption spectrum with incident illumination by varying charge density. As a result, the elevated steady-state temperature of the nanostructure induced by plasmonic absorption is further increased by a small amount. Here, we study in detail the thermodynamic theory underlying the plasmoelectric effect by analyzing a simplified model system consisting of a single silver nanoparticle. We find that surface potentials as large as 473 mV are induced under 100 W/m^2^ monochromatic illumination, as a result of a 11 mK increases in the steady-state temperature of the nanoparticle. Furthermore, we discuss the applicability of this analysis for realistic experimental geometries, and show that this effect is generic for optical structures in which the resonance is linked to the charge density.

Surface plasmons are collective oscillations of charge density in metal nanostructures during optical excitation. Recently, there has been significant interest in the relationship between plasmonic and electrical phenomena^1^,^2^,^3^,^4^,^5^,^6^,^7^,^8^,^9^,^10^,^11^. For example, localized plasmon resonances have been shown to influence the photoconductivity of films of metal nanoparticles coated with self-assembled monolayers^12^,^13^, and plasmon-induced hot electrons have been shown to generate macroscopic currents in plasmonic energy conversion devices^6^. Furthermore, it has been demonstrated that electrostatic charging influences the plasmon resonance frequency^11^. The plasmonic response of metal nanoparticles is determined by geometry, dielectric surrounding and material properties^14^. In the visible spectral range the dielectric function of the metal can often be well described by a Drude model. In this model the plasmon resonance frequency depends on electron density, *n*_*e*_, via the bulk plasma frequency *ω*_*p*_ 15,16:


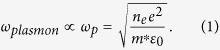


Here, *e* is the electron charge, m* is the effective electron mass^17^, and *ε*_0_ is the free-space permittivity. Equation 1 shows that the plasmon resonance frequency is directly linked to the electron density *n*_*e*_, and thus may be tuned by varying *n*_*e*_. Indeed, recent experiments have demonstrated spectral shifts up to 43 nm in the plasmon scattering spectrum of Au nanoparticles by adding chemical reductants to the nanoparticle solution that induce a negative charge at the surface of the particles^15^. Also, spectral shifts up to 11 nm have been demonstrated using electrostatic biasing of Au nanoparticles in electrochemical cells^18^,^19^,^20^. Electron density changes as high as 11% relative to the electron density in the uncharged state were observed. These experiments show a clear relationship between the optical properties of metal nanoparticles and their electron density, as predicted by the Drude model.

Recently, we have demonstrated a *plasmoelectric effect* in which an optically induced electrostatic potential is generated if a metal nanostructure is illuminated off-resonance^21^. This effect is driven by a thermodynamic increase in entropy, which originates from the dependence of the plasmon resonance on electron density. Here, we discuss in detail the thermodynamic theory underlying the plasmoelectric effect. We first explore a simplified model consisting of a single plasmonic nanoparticle in vacuum to describe the fundamental theory, and subsequently discuss implications for realistic experimental geometries.

## Plasmoelectric effect

**Figure 1:**
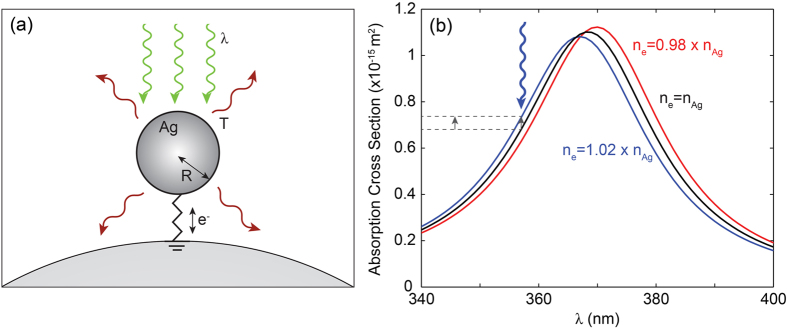
(**a**) Schematic of the model: a silver nanoparticle with radius *R* in vacuum is electrically connected to ground. Monochromatic illumination with wavelength *λ* and intensity *I*_*λ*_ is absorbed and excites a plasmon resonance, heating the particle to temperature *T*. Thermal radiation is the only power loss channel. (**b**) Calculated absorption cross section for a Ag nanoparticle (*R* = 10 nm) in vacuum with an electron density 2% higher (blue), lower (red), or equal to that of neutral silver (black). Under off-resonance monochromatic illumination, changes in the electron density increase the absorption cross section (gray arrows).

To introduce the plasmoelectric effect, we explore the reversed phenomenon of electrostatic modulation of a plasmon resonance via the dependence on electron density^18^,^19^,^20^. Consider a silver nanoparticle in vacuum with radius, *R*, that is electrically connected to ground (Fig. 1a). Thermal fluctuations will cause electrons to randomly enter or exit the nanoparticle, thereby inducing minute fluctuations of electron density. In the dark and under equilibrium conditions the net electron flux is zero. For the analysis here, we first assume radiation provides the only pathway for transferring thermal energy in or out of the particle. Now, consider if the nanoparticle is illuminated with monochromatic radiation at wavelength *λ* and intensity *I*_*λ*_ that is blue-shifted with respect to the plasmon resonance of the nanoparticle. Small thermal fluctuations in electron density will cause small fluctuations in the absorption cross section at the illumination wavelength *C*_*abs*_(*λ*) (Fig. 1b). Since optical absorption induces heating of the nanoparticle, these small fluctuations result in small changes of the nanoparticle temperature *T* and, in turn, thermodynamic quantities such as entropy and internal energy. For the situation with blue-shifted incident light, thermal fluctuations that add electrons to the nanoparticle thus increase temperature and entropy, which implies that spontaneous *increases* of charge density are thermodynamically favored. Vice versa, if the incident light is red-shifted with respect to the plasmon resonance, a *decrease* of electron density increases entropy. At the same time, Coulombic interactions induce a counteracting force against charging of the nanoparticle. To calculate the charge of the nanoparticle during illumination in steady state, we consider the thermodynamic free energy of the system *F*_*tot*_, and minimize it with respect to the number of electrons in the nanoparticle *N*.


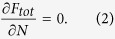


As the nanoparticle temperature *T* also depends on *N*, Eq. 2 can be expanded as


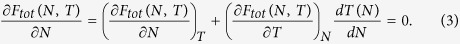


Next, we recognize the general definition for the chemical potential *μ*


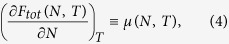


and for the entropy *S*


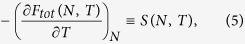


so that Eq. 3 can be written as


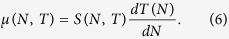


Equation 6 demonstrates the essence of the plasmoelectric effect: a non-zero electrochemical potential results from the dependence of temperature on electron number in a plasmonic nanoparticle. This analysis is based upon the minimization of the thermodynamic free energy of the system, which we argue to be accurate for the steady-state conditions considered here (see Discussion section). Note that electron density *n*_*e*_ and number of electrons in the nanoparticle *N* are related by *N* = *n*_*e*_*V*, where *V* is the (constant) particle volume.

The amplitude and spectrum of the plasmonically induced electrochemical potential (i.e. the *plasmoelectric potential, V*_*PE*_) can be calculated, fully analytically, in five steps. First, we use Mie theory to calculate the dependence of the absorption cross section *C*_*abs*_(*n*_*e*_, *λ*) on electron density. Second, we use this cross section in a steady-state power balance to calculate the particle temperature *T*(*N*, *λ*). The derivative of this function with respect to *N* for fixed *λ* yields *dT*(*N*)/*dN* in Eq. 6. Third, we determine the total free energy of the system using well-known definitions for the free energy of a free electron gas and crystal lattice. Fourth, we use Eqs 4 and 5 to derive definitions for *μ*(*N*, *T*) and *S*(*N*, *T*) in Eq. 6. Finally, Eq. 6 can be solved for *N* for a range of *λ* to determine *N*(*λ*), from which we calculate the surface potential *V*_*PE*_(*λ*). In the remainder of the paper, we will discuss each step in detail and finally explore the influence of geometrical parameters and illumination conditions on *V*_*PE*_.

## Absorption cross section and steady-state temperature

We use Mie theory^22^ to calculate the electron density dependence of the absorption cross section *C*_*abs*_(*n*_*e*_, *λ*). For the dielectric function of Ag, we apply a sixth-order multiple Lorentz-Drude fit (see Supplemental Information) to data from Palik^23^, analogous to the method outlined by Rakic *et al*.^24^. This dielectric function depends explicitly on the electron density *n*_*e*_ through the bulk plasma frequency, as described in Eq. 1. This method has been demonstrated to accurately describe charge carrier-dependent shifts of the plasmon resonance in both metal nanoparticles^15^,^19^, and doped semiconductor quantum dots^16^,^25^.

**Figure 2:**
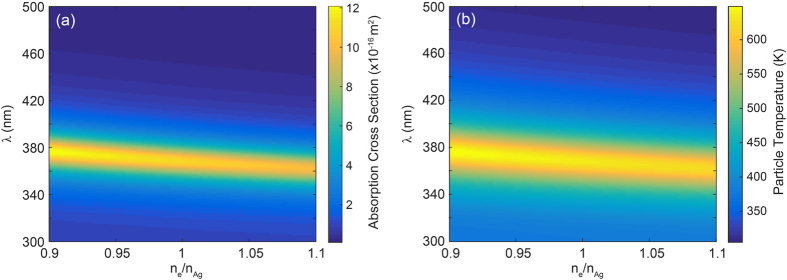
(**a**) Calculated electron density dependent absorption cross section (color) for a Ag nanoparticle (*R* = 10 nm) in vacuum, for 0.9 ≤ *n*_*e*_/*n*_*Ag*_ ≤ 1.1. (**b**) Steady-state particle temperature (color) for *I*_*λ*_ = 100 W/m^2^ (monochromatic).

Figure 2a shows the calculated absorption cross section for a Ag particle (*R* = 10 nm) in vacuum. A clear peak in absorption as a result of the lowest order dipolar plasmon resonance can be observed around *λ* = 367 nm for the neutral particle; it shows a monotonic blue-shift with increasing electron density, as expected from Eq. 1. Next, we use the results from Fig. 2a to calculate the steady-state particle temperature *T*(*n*_*e*_, *λ*). Note that for the illumination powers considered here, the electron temperature equals the phonon temperature due to the fast electronic relaxation rate and electron-phonon coupling rate in a metal^26^:


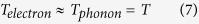


In steady state, the power absorbed by the particle is balanced by the power going out:


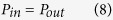


The absorbed power includes both the absorbed monochromatic radiation and the ambient thermal background radiation (*T*_*amb*_ = 293 K), which is given by the Stefan-Boltzmann law:


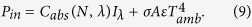


Here, *σ* is the Stefan-Boltzmann constant, *A* is the particle surface area, and *ε* is the emissivity. The nanoparticle emissivity is taken to be equal to that of bulk silver, *ε* = 0.01, as no modulation to the bulk properties is predicted by Mie theory in the spectral range of thermal radiation. Indeed, despite that plasmonic nanoparticles can be strong absorbers at wavelengths close the the plasmon resonance, the low emissivity of the bulk material results in a low IR emissivity^27^. In vacuum, the only power loss channel is through thermal radiation:


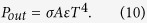


Using Eqs 9 and 10 to solve Eq. 8 for *T* yields the steady-state particle temperature


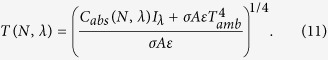


Figure 2b shows the calculated steady-state particle temperature for *I*_*λ*_ = 100 W/m^2^. A peak temperature of 634 K is observed for a neutral particle at resonance, and the temperature profile clearly follows the absorption profile as expected from Eq. 11. Finally, converting the x-axis from *n*_*e*_ to *N* and taking the derivative with respect to *N* at a given wavelength yields *dT*(*N*)/*dN*, the last term in Eq. 6.

## Free energy calculations

The free energy of the system can be obtained by considering the separate contributions of the electrons *F*_*e*_, and phonons, *F*_*p*_ in the nanoparticle as well as those of the substrate (the electrical ground, subscript *s*):


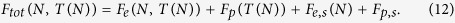


Note that *F*_*e*,*s*_ is a function of *N* through the conservation of total charge *N*_*tot*_ = *N* + *N*_*s*_: i.e. if *N* > *N*_0_, with *N*_0_ the number of electrons in a neutral nanoparticle, the chemical and electrostatic potential of the substrate also changes (*N*_*s*_ < *N*_0,*s*_). *F*_*e*_ is composed of the electron chemical potential *μ*_*e*_, and the electrostatic potential, Φ:


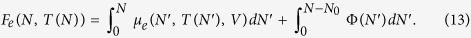


Here, *μ*_*e*_ is the temperature dependent chemical potential of a free electron gas^28^:


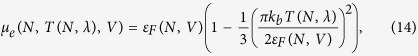


where *k*_*b*_ is Boltzmann’s constant, and *ε*_*F*_ is the Fermi energy


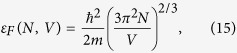


with 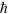 the reduced Planck constant. The temperature-dependent term in Eq. 14 is <0.01% at the temperatures considered here^28^. The electrostatic potential is easily obtained from the self-capacitance of a sphere *c* = 4*πRε*_0_*ε*_*m*_, where *ε*_*m*_ is the relative permittivity of the surrounding medium. The work *W* required to charge a capacitor with charge *Q* = *N*′*e* is


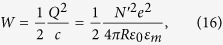


where *e* = −1.602 × 10^−19^ C is the electron charge, so that


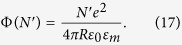


*F*_*p*_ is expressed in terms of the speed of sound in the particle *v*_*s*_, as obtained from the high-temperature classical limit of the Debye model^29^,^30^:


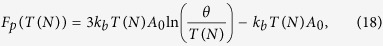


where *A*_0_ is the number of atoms in the nanoparticle (equal to *N*_0_ for Ag), and *θ* the Debye temperature^29^:


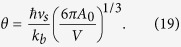


We use *θ*_*Ag*_ = 215 K^30^.

To quantify the free energy of the substrate, we assume the substrate (electrical ground) is a macroscopic silver sphere with radius *R*_*s*_ and volume *V*_*s*_ at fixed ambient temperature (*T*_*amb*_), containing *A*_0,*s*_ atoms and *N*_0,*s*_ electrons in a neutral state. If the number of electrons transferred from the substrate to the nanoparticle is *N* − *N*_0_, *F*_*e*,*s*_ and *F*_*p*,*s*_ are given by


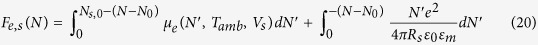


and


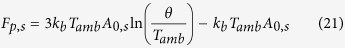


respectively.

## Electrochemical potential and entropy

Equation 4 is applied to Eq. 12 to obtain the electrochemical potential. Starting with *F*_*e*_(*N*, *T*(*N*)), we use


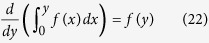


to get


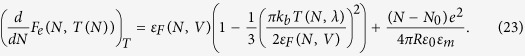


Next, since *F*_*p*_ does not depend on *N* for constant *T*,


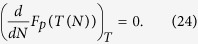


Using the fact that the substrate is macroscopic (e.g. *R*_*s*_ > 1 cm, 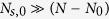 and *c*_*s*_ ≫ *c*), the effect of electrostatic charging of the substrate is negligible and the derivative of *F*_*e*,*s*_ can be simplified to


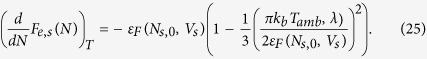


Note that Eq. 25 simplifies to −*ε*_*F*_(*N*_0_, *V*) if the temperature dependence is neglected. Finally,


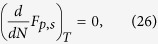


such that the complete definition for *μ*(*N*, *T*(*N*)) in Eq. 6 is given by:


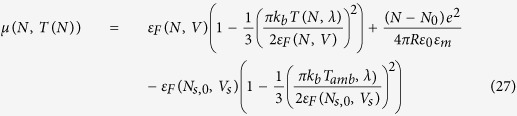


Next, we obtain a definition for the entropy of the system by applying Eq. 5 to Eq. 12. Since the electrostatic potential does not depend on *T*,


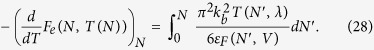


The nanoparticle phonon entropy is easily obtained as


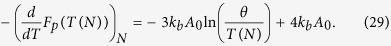


Finally, since the grounded substrate has constant temperature *T*_*s*_ = *T*_*amb*_,


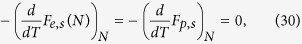


such that


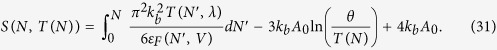


## Master equation

**Figure 3:**
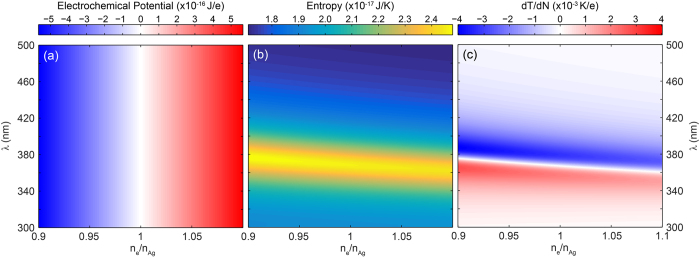
Electrochemical potential *μ* (**a**), entropy *S* (**b**), and plasmonically induced temperature dependence *dT*/*dN* (**c**) for a Ag particle (*R* = 10 nm) in vacuum under 100 W/m^2^ monochromatic illumination as a function of electron density and wavelength.

Based of the definitions of *μ* (Eq. 27), *S* (Eq. 31) and *dT*/*dN* (Eq. 11 and Fig. 2b), the free energy minimization can now be applied to find the steady-state (time-averaged) charge state of the nanoparticle for each illumination wavelength *λ*. As it is instructive to explore the terms in Eq. 6 individually we first calculate *μ, S* and *dT*/*dN* for the 20 nm Ag nanoparticle model system. Figure 3 shows the electrochemical potential (a), entropy (b) and plasmonically induced temperature dependence *dT*/*dN* (c) for *I*_*λ*_ = 100 W/m^2^.

The electrochemical potential (Fig. 3a) shows a monotonic, wavelength independent increase with electron density. Comparing this trend with the particle temperature in Fig. 2b, which shows a clear wavelength dependence as a result of the resonance, indicates that the temperature dependence of *μ* is indeed negligible. Furthermore, from the linear increase with *n*_*e*_ (and thus *N*), is it clear that the electrostatic Coulombic interaction dominates over the Fermi energy, which scales with ∝*N *^2/3^ (see Eq. 15). Indeed, comparing the increase in Fermi energy (Eq. 15) and electrostatic potential (Eq. 17) as a result of the addition of a single electron to the neutral particle shows that the effect on the electrostatic potential is 4 orders of magnitude larger than that on *ε*_*F*_.

Equation 31 shows a dependence of *S* on *N* through the first and second term. The first term corresponds to the entropy of the electron gas, which is negligible compared to the lattice entropy. The second term scales with ∝log(*T*(*N*)). Finally, the third term in Eq. 31 is constant and only determined by the number of atoms *A*_0_ in the nanoparticle. Hence, the entropy will be constant with a small modulation that scales logarithmically with the particle temperature (Fig. 2b). This is confirmed by the trend in Fig. 3b, which clearly follows that of *T*. The entropy of the system is thus clearly dominated by the entropy of the lattice.

Figure 3c shows *dT*(*N*)/*dN*, calculated from Fig. 2b. Three distinct features can be observed. First, *dT*/*dN* = 0 at the resonance wavelength of the neutral particle. Since *S* × *dT*/*dN* is the thermodynamic driving force for the plasmoelectric potential, this implies that *V*_*PE*_ = 0 at the resonance wavelength of the neutral particle. Second, it is important to realize that *V*_*PE*_ is calculated for fixed incident wavelength. Hence, it is the horizontal cross cut through Fig. 3c that determines the magnitude of the thermodynamic driving force. Third, with this in mind it is clear from Fig. 3c that it is beneficial to have plasmonic resonances with high quality factor (*Q*), but it is the sensitivity of the resonance wavelength to the electron density that determines the magnitude of *V*_*PE*_ in the end. This sensitivity is characterized by the slope of the white line in Fig. 3c.

Equation 6 shows that the free energy minimum can be found by equating *μ* to *S* × *dT*/*dN*, corresponding to where data in Fig. 3a equals the product of data in Fig. 3b,c. Considering the order of magnitude of the different terms in Fig. 3b,c shows that *S* × *dT*/*dN* < 9 × 10^−20^ J/e. Comparing this with the magnitude of the electrostatic potential in Fig. 3a (up to 5 × 10^−16^ J/e) shows that the free energy minimum will occur very close to charge neutrality. Indeed, adding a single electron to a 20 nm nanoparticle leads to a change in surface potential of 144 mV.

Finally, note that the master equation can be greatly simplified. Neglecting the temperature dependence in the chemical potential (Eq. 27), using that 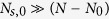, and neglecting the electron entropy (Eq. 28), simplifies the equation to


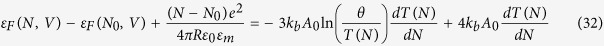


with <0.25% error in the maximum calculated potential. All results in this work are calculated using the full equation rather than the simplified one.

## Results

**Figure 4:**
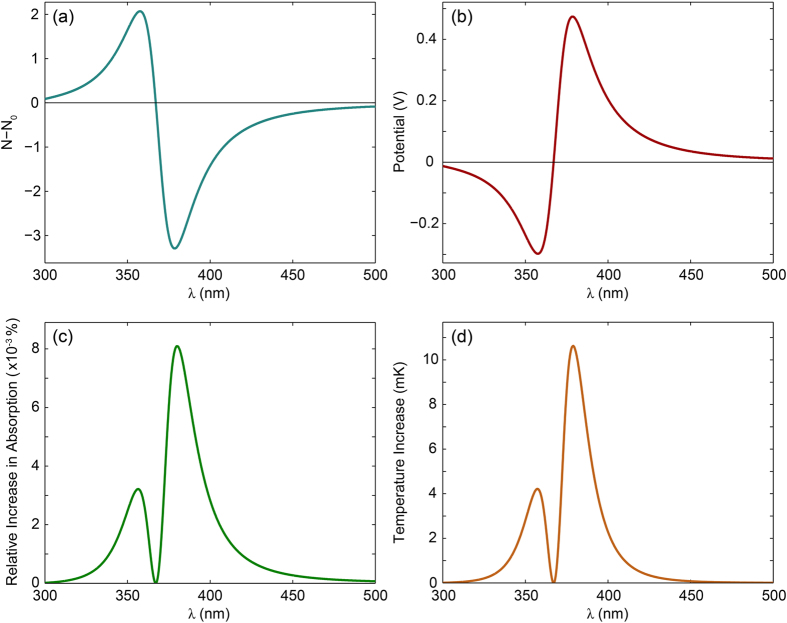
Steady-state configuration as a function of wavelength of a 20 nm Ag particle in vacuum under *I*_*λ*_ = 100 W/m^2^ monochromatic illumination. (**a**) Time-averaged number of electrons transferred from the substrate into the nanoparticle. (**b**) Induced plasmoelectric surface potential. (**c**) Relative increase in absorption as result of plasmoelectric effect. (**d**) Corresponding increase in particle temperature compared with a particle that remains neutral.

Solving Eq. 6 for *N* for a range of *λ* yields *N*(*λ*). Figure 4a shows the time-averaged number of electrons transferred from the substrate to the nanoparticle (*N* − *N*_0_) as a function of wavelength for a 20 nm Ag particle (*I*_*λ*_ = 100 W/m^2^). Three trends are worth noting in Fig. 4a. First, a clear bisignated signal is observed which is positive on the blue side and negative on the red side of the neutral particle resonance wavelength (*λ*_*res*_ = 367 nm). The shape of the signal clearly indicates the trend of the plasmoelectric effect: electrons are added to the nanoparticle for *λ* < *λ*_*res*_ to blue-shift the absorption resonance and thereby increase the entropy. Vice versa, electrons are removed from the particle for *λ* > *λ*_*res*_ to red-shift the absorption resonance. Second, the signal is maximized when *S* × *dT*/*dN* is maximum, which roughly corresponds to where the slope in the absorption spectrum is maximum. Third, the signal is asymmetric: the amplitude of the signal is larger above *λ*_*res*_ than below. This is caused by: 1) the non-resonant intrinsic interband absorption in the silver, and 2) the fact that *C*_*abs*_ ∝ *λ*^2^ according to Mie theory.

Three observables can be derived from *N*(*λ*). First, a static surface potential will be induced on the nanoparticle if *N* ≠ *N*_0_, which is defined by both the difference in Fermi energy as well as the potential due to electrostatic charging of the self-capacitance:


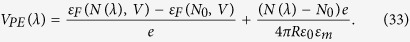


Second, the plasmoelectric effect is driven by an increase in absorption as a result of electron transfer. Therefore, the relative increase in absorption compared to a neutral particle can be calculated as


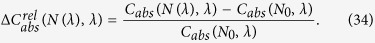


Finally, the increase in absorption results in an increase in the particle temperature:


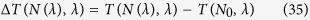


Figure 4b–d show the calculated plasmoelectric potential (b), relative increase in absorption (c) and corresponding increase in temperature (d) as a result of the plasmoelectric effect. *V*_*PE*_ scales with −*N* since the electrostatic potential dominates the amplitude of the potential. Therefore, the potential has the same but negative trend as Fig. 4a. As a result of the small capacitance of the nanoparticle the potential reaches several 100 mV for the transfer of only a few electrons.

Due to the small number of electrons transferred, the spectral shift of the plasmon resonance is very small. Therefore, the relative increase in absorption is limited to a maximum of 8 × 10^−3^%, corresponding to a maximum increase in temperature due to the charge transfer of ~11 mK. Note that this small increase in temperature is with respect to that of a neutral particle under steady-state illumination. Starting from a neutral particle in the dark, *T* increases from *T*_*amb*_ to *T* ~ 630 K due to steady-state absorption of monochromatic radiation. Then, by adding or subtracting on average 2–3 electrons (Fig. 4a) the temperature is further increased by ~11 mK (Fig. 4d). Finally, unlike the potential, the increase in absorption and temperature is always positive due to the requirement of entropy maximization. Interestingly, the increase in absorption and temperature vanishes at *λ*_*res*_, which distinguishes the plasmoelectric effect from e.g. the thermoelectric effect, which would be maximum at *λ* = *λ*_*res*_ due to the maximum induced temperature at that wavelength.

## Illumination conditions

The intrinsic thermodynamic nature (entropy maximization and free energy minimization) of the plasmoelectric effect indicates that it is not a linear optical phenomenon. However, the amplitude of the potential is not directly described by higher-order non-linear susceptibility terms *χ*^(*i*)^, as is, for example, plasmon-enhanced second harmonic generation^31^. The scaling of *V*_*PE*_ with *I*_*λ*_ is determined by the steady-state particle temperature, and thereby the power gain and loss channels available to the system. For a nanoparticle in vacuum, *P*_*in*_ is linear with *I*_*λ*_. However *P*_*out*_ is limited to thermal radiation, which scales with ∝*T*^4^ (Eq. 10). Therefore, *V*_*PE*_ initially shows a rapid increase with increasing *I*_*λ*_, but saturates as *P*_*out*_ increases ∝*T*^4^. To demonstrate this, we calculate the induced potential for the same 20 nm Ag nanoparticle as considered in Figs 2, 3, 4, but now for 0 ≤ *I*_*λ*_ ≤ 250 W/m^2^.

**Figure 5:**
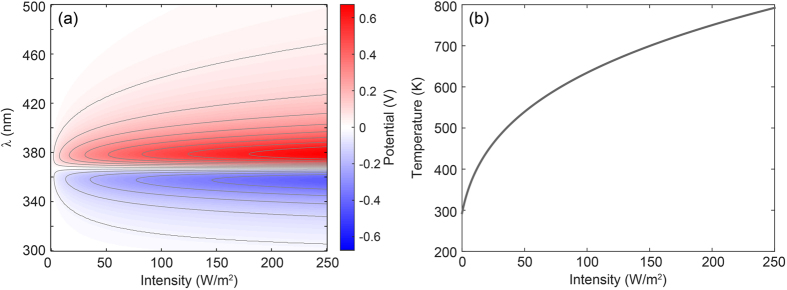
(**a**) Plasmoelectric potential on a 20 nm Ag sphere in vacuum as a function of monochromatic illumination intensity and wavelength. The gray lines show iso-potential lines. (**b**) Corresponding maximum temperature of the nanoparticle.

Figure 5 shows the calculated *V*_*PE*_ (a) and maximum particle temperature (b) as a function of *I*_*λ*_. Both the potential and the steady-state temperature show a strong increase at low intensities, which is clearly visible from the closely spaced iso-potential lines (a). The saturation as a result of the rapid increase in thermal radiation is also clearly visible in both figures. Furthermore, the iso-potential lines in Fig. 5a show a strong spectral shift away from *λ*_*res*_ with increasing *I*_*λ*_. As a result, the thermodynamic driving force increases with *I*_*λ*_ such that *dT*/*dN* increases at the shoulders of the resonance spectrum, thereby increasing the spectral bandwidth of the plasmoelectric effect.

Note that for realistic experimental geometries (i.e. particles on a substrate in air), thermal diffusion and convection rather than radiation dominate the thermal response, thereby changing the proportionality of *V*_*PE*_ ∝ *I*_*λ*_. The thermal power balance also dictates the maximum intensity that can be used in experiments, since the power loss channels determine the damage threshold of the resonant structure.

## Particle size

**Figure 6:**
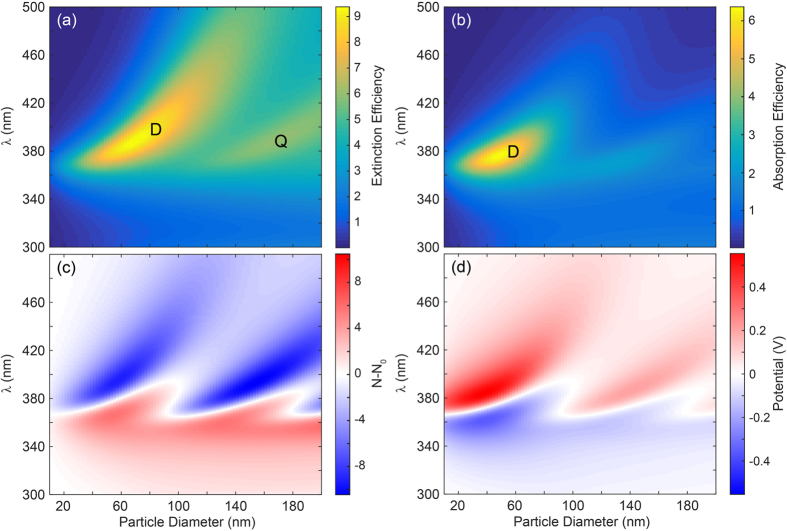
(**a**) Calculated extinction efficiency (*σ*_*ext*_/*σ*_*geo*_) for a Ag sphere in vacuum as a function of particle diameter. The dipolar (D) and quadrupolar (Q) resonant modes are labeled accordingly. (**b**) Calculated absorption efficiency (*σ*_*abs*_/*σ*_*geo*_) showing the increase in albedo for larger particle diameters. (**c**) Transferred average number of electrons (**c**) and induced plasmoelectric potential (**d**) as a result of the dipolar and quadrupolar resonance under 100 W/m^2^ monochromatic illumination.

Next, we study the influence of particle size on the plasmoelectric potential. The localized plasmon resonance is strongly sensitive to the particle geometry^14^, and shows a red-shift with increasing particle diameter^32^. Furthermore, the electrostatic capacitance of the nanoparticle scales with *R*, such that the number of electrons transferred to/from the nanoparticle will increase for a given potential. Figure 6a shows the calculated extinction efficiency, which is defined as *σ*_*ext*_/*σ*_*geo*_, where *σ*_*geo*_ = *πR*^2^ is the geometrical cross section of the nanoparticle. The dipolar (D) resonance, observed at *λ*_*res*_ = 367 nm for the 20 nm particle (Fig. 1b), shows a clear red-shift and increase in extinction efficiency with increasing diameter. For *d* > 100 nm, a higher order quadrupolar resonance (Q) occurs, which also red-shifts with increasing particle diameter. A maximum extinction efficiency of 9.38 is observed for *d* = 68 nm, showing the strongly resonant behavior of the nanoparticle. Comparing the trends in Fig. 6a with the 20 nm particle considered in Figs 1, 2, 3, 4, 5 could suggest that larger plasmoelectric potentials can be obtained for *d* ~ 70 nm than for *d* = 20 nm. However, the albedo (*σ*_*scat*_/*σ*_*ext*_) of the resonance also increases with particle size^33^. Hence, a smaller fraction of the extinction is due to absorption by the nanoparticle. Figure 6b shows the absorption efficiency (*σ*_*abs*_/*σ*_*geo*_) as a function of particle size. It shows both the dipolar and quadrupolar resonance, and a strong reduction in the absorption efficiency is observed for *d* > 70 nm as a result of the increased scattering rate. An optimum absorption efficiency of 6.37 is observed for *d* = 48 nm. Note that this is not necessarily the optimum geometry for the plasmoelectric effect either, since it is the increase in absorption per added electron (*dT*/*dN*), which depends on radius, that determines the magnitude of the potential rather than the absolute absorption.

The average number of electrons transferred to the nanoparticle (Fig. 6c) shows a general positive trend for *λ* < *λ*_*res*_, and a negative trend for *λ* > *λ*_*res*_, for all particle sizes. The zero-point crossing line (i.e. where *N* − *N*_0_ = 0) clearly follows the dipolar and quadrupolar resonances observed in Fig. 6b, indicating that the plasmoelectric effect is not limited to the dipolar nature of the lowest order plasmon resonance. Furthermore, a significant increase in *N* − *N*_0_ is observed for increasing particle diameter. Since *Ne* = *cV*_*PE*_ and *c* ∝ *R*, the number of charges strongly increases as a result of the reduced electrostatic repulsion on larger particles. Note that the capacitance, and thereby the number of transferred number of electrons can be drastically enhanced by embedding the nanoparticle in a water-based electrolyte, as a result of the large static dielectric constant of water and the double-layer screening by counter ions^19^. Finally, the charge transfer induced by the dipolar and quadrupolar resonance counteract each other for 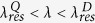. The dipolar resonance induces an increase in charge density, whereas the quadrupolar resonance induces a reduction. This balance causes a “dead” region in between the two resonances around *d* = 100 nm.

Next, we calculate the potential induced on the nanoparticle by the charge transfer (Fig. 6d). The potential follows roughly the same trends as the charge transfer (Fig. 6c), except that a strong reduction is observed with increasing particle diameter. Comparing Fig. 6d with Fig. 6b shows why: the absorption efficiency shows a strong decrease with increasing particle size, suggesting that the increase in *N* − *N*_0_ in Fig. 6c is dominated by the increase in *c*. A maximum potential of 554 mV is observed for *d* = 38 nm and *λ* = 384 nm. Figure 6c,d show that the particle geometry can be tuned to optimize the plasmoelectric effect for maximum charge transfer (large particle) or maximum potential (small particle).

## Thermodynamic potential

**Figure 7:**
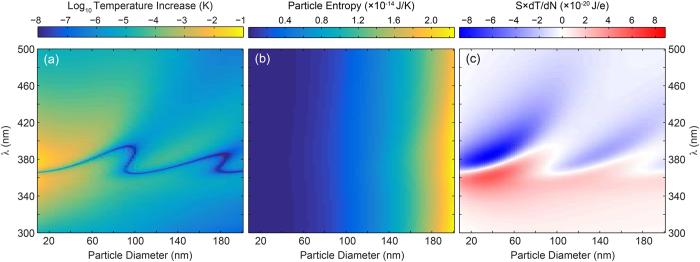
(**a**) Log_10_ of increase in temperature (in K, color) as a function of particle diameter and wavelength for *I*_*λ*_ = 100 W/m^2^, showing a rapid decrease with increasing particle size. (**b**) Entropy of the nanoparticle (for *N* = *N*_0_), showing a strong increase with increasing particle size. (**c**) Thermodynamic potential *S* × *dT*/*dN* (evaluated at *N* = *N*_0_) driving the plasmoelectric effect.

Figure 6 shows that the absorption profile (Fig. 6b) does not unambiguously predict the plasmoelectric potential (Fig. 6d); i.e. the potential is not maximum where absorption is maximum. To explain this we consider *S* and *dT*/*dN* separately as a function of particle size. Similar to Fig. 4c, the increase in temperature as a result of the plasmoelectric effect can be studied by considering the intuitive quantity 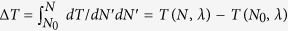. Figure 7a shows Δ*T* (log_10_, color) obtained from the results of Fig. 6. A small overall increase in temperature is observed for wavelengths close to the *λ*_*res*_ (corresponding to the dark blue line). The oscillations in the dark blue line with increasing particle diameter correspond to the subsequent transitions from dipole to quadrupole, and quadrupole to octopole plasmon resonance modes dominating the plasmoelectric effect, respectively. Although the increase in temperature is significant for small particles (up to 25.4 mK for *d* = 10 nm), it rapidly decreases with increasing particle diameter. In fact, for *d* = 200 nm Δ*T* peaks at only 6 *μ*K, which indicates a three orders of magnitude reduction in Δ*T* compared to small particles. Yet still, a significant potential 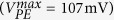 is observed for such large particles (Fig. 6d).

To understand why, Fig. 7b shows the entropy of a neutral nanoparticle *S*(*N*_0_) (color) as a function of particle size and incident wavelength. Note that *S*(*N*) ≈ *S*(*N*_0_) due to the logarithmic dependence on the very small variations in *T*(*N*). The particle entropy shows a drastic increase with particle size, as *A*_0_ ∝ *R*^3^ (Eq. 31). Indeed, *S* increases from ~10^−17^ J/K for *d* = 20 nm with three orders of magnitude up to ~10^−14^ J/K for *d* = 200 nm. Note that the small variation of *S* with *λ* originates from the dependence of the temperature on *N*: *S*(*T*(*N*)), see Eq. 31 and Fig. 3b.

Finally, Fig. 7c show 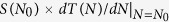, which corresponds to the thermodynamic potential experienced by a neutral particle under monochromatic illumination. Figure 7a,b show that for larger particles the increase in temperature drops by three order of magnitude, whereas the entropy increases by three orders of magnitude. As a result, the product (Fig. 7c) has the same order of magnitude for the entire range of particle diameters. Indeed, comparing Fig. 7c with Fig. 6d shows that the product *S* × *dT*/*dN* scales with the calculated potential (converted from J/e to V). This example demonstrates that for large systems, the increase in temperature as a result of the plasmoelectric effect can be very small. However, due to the large entropy of the system, the energetic pay-off of this small increase in temperature is sufficiently large to induce significant surface potentials. Thus more realistic experimental geometries can still be expected to exhibit appreciable plasmoelectric potentials, e.g. particles on a substrate in air, where thermal convection and conduction may significantly lower the temperature obtained under illumination.

## Discussion

### Generalization

The plasmoelectric effect is not limited to plasmonic resonators. It is generic for a resonant optical cavity that tends to spectrally align its resonance with the pump light to optimize absorption. Conditions to achieve this are: First, the system has an optical resonance that exhibits non-radiative losses, i.e. absorption. The absorption generates heat and thereby entropy, which is the underlying thermodynamic driving force. Second, there exist a feedback mechanism between the resonance frequency and the electron density of the structure. Essential for the bisignated signal is that the feedback mechanism works in both ways: e.g. an increase (decrease) in electron density causes a blue- (red) shift of the optical resonance. Third, the system is electrically connected to ground or a (large) electron bath, which allows exchange of electrons with the resonator. Note that the simplified model system considered here neglects the influence of such an electrical connection to ground on the dielectric environment of the nanoparticle. In reality, the presence of e.g. a conductive substrate may red-shift the resonance wavelength and give rise to spectral broadening. Although these effects may change the spectral shape and amplitude of the plasmoelectric potential, the physical mechanism governing the plasmoelectric effect remains unaltered.

These requirements can also be met with non-plasmonic resonators such as for example whispering gallery cavities based on doped semiconductor or transparent conductive oxides structures, which can be heavily doped^34^. Significant changes in the refractive index of such materials have been demonstrated through electrical gating^35^, and electrically tunable resonances in these devices using this effect have been realized^36^. This offers great potential for the use of these structures in plasmoelectric circuitry.

### Free energy minimization for local-equilibrium systems

The thermodynamic analysis discussed in section is based upon the minimization of the thermodynamic free energy of the system. By definition, this analysis is strictly warranted when describing the equilibrium state of a closed system with fixed total energy^37^. Although the system we model here does not approach a true thermodynamic equilibrium, i.e. we do not consider a closed system which includes the thermodynamic state of the optical source, we argue that free-energy minimization still gives an accurate description. The steady-state power balance dictates a well defined particle temperature: in Fig. 2b, a maximum temperature of 634 K was observed. For the optical intensities considered, the electronic and phononic distribution of the particle is not driven to a non-thermal state, but is expected to be well described by conventional Fermi-Dirac and Boltzmann statistics, respectively^26^. We stress that similar arguments hold for the well-established detailed-balance calculation of the limiting efficiency of a photovoltaic cell^38^, which also considers a steady-state system that is not in thermal equilibrium with a source but assumes the validity of a thermal distribution among the excited carriers in the conduction band.

## Conclusions

In conclusion, we present a thermodynamic theory of the plasmoelectric effect using a model system composed of a 20 nm Ag sphere in vacuum. We show that minimization of the thermodynamic free energy of the system leads to an electrostatic surface potential on a resonant metal nanoparticle, driven by the increase in absorption - and thereby entropy production - as a result of electron injection. The spectral shift induced by the electron injection gives rise to a small increase in the steady-state temperature of the particle, in addition to the elevated temperature obtained by a neutral particle as a result of plasmonic absorption. We find that for our model system, potentials up to 473 mV are induced under 100 W/m^2^ monochromatic illumination as a result of an 11 mK increase in the steady-state particle temperature. Furthermore, the plasmoelectric potential is found to be non-linear with the illumination intensity, as the amplitude of the potential is dictated by the steady-state thermal balance. We determine how the plasmoelectric potential scales with particle size, and find that for large systems, minute increases in temperature can induce significant surface potentials as a result of the large entropic pay-off. Finally, we discuss the conditions required for manifestation of the plasmoelectric effect, and predict that the effect is generic for any resonant system in which optical absorption and charge density are coupled.

## Additional Information

**How to cite this article**: van de Groep, J. *et al*. Thermodynamic theory of the plasmoelectric effect. *Sci. Rep.* **6**, 23283; doi: 10.1038/srep23283 (2016).

## Supplementary Material

Supplementary Information
