# Supplementary material for: Thermodynamic theory of the plasmoelectric effect
Source: Sci Rep. 2016 Mar 18;6:23283. doi: 10.1038/srep23283 (PMC4796873; doi:10.1038/srep23283)
Supplement: Supplementary Information [file srep23283-s1.pdf]

Thermodynamic theory of the plasmoelectric effect - Supplemental Information

J. van de Groep[1], M.T. Sheldon[2,3], H.A. Atwater[3], and A. Polman[1,*]

[1]Center for Nanophotonics, FOM Institute AMOLF, Science Park 104, 1098 XG Amsterdam, Netherlands
[2]Department of Chemistry, Texas A&M University, College Station, TX 77843, USA
[3]Thomas J. Watson Laboratories of Applied Physics, California Institute of Technology, MC 128–95, Pasadena, CA 91125, USA
[*]polman@amolf.nl

ABSTRACT

This document contains Supplemental Information for “Thermodynamic theory of the plasmoelectric effect”, describing the complex dielectric function of Ag.

1 Complex dielectric function of Ag

We use a sixth-order Lorentz-Drude fit to the data from Palik,[1] using the approach outlined by Rakic et al.[2] The detailed procedure and the fit parameters are obtained from ref.[3] In short, the model used is

$$\varepsilon_{LD}(\omega) = 1 - \frac{f_0\omega_p^2}{\omega^2 + i\Gamma_0\omega} + \sum_{j=1}^{5} \frac{f_j\omega_p^2}{\omega_j^2 - \omega^2 - i\Gamma_j\omega}, \tag{1}$$

where f_0 and Γ_0 are the amplitude and damping rate of the Drude (intraband) term, describing the free-electron contribution to the dielectric function. Similarly, f_j, ω_j and Γ_j are the amplitude, resonance frequency, and damping rate of the j-th Lorentz oscillator describing the interband part of the dielectric function. The fitting parameters used are:

Term	f	ω_p (rad/s)	ω_j (rad/s)	Γ_j (rad/s)
$j=0$	0.845	13.69×10^{15}	0.000	0.0729×10^{15}
$j=1$	0.065	13.69×10^{15}	1.240×10^{15}	5.904×10^{15}
$j=2$	0.124	13.69×10^{15}	6.808×10^{15}	0.6867×10^{15}
$j=3$	0.011	13.69×10^{15}	12.44×10^{15}	0.0988×10^{15}
$j=4$	0.840	13.69×10^{15}	13.80×10^{15}	1.392×10^{15}
$j=5$	5.646	13.69×10^{15}	30.83×10^{15}	3.675×10^{15}

Table 1. Fitting parameters used for the Lorentz-Drude fit in Eq. 1.

References

1. Palik, E. D. *Handbook of Optical Constants of Solids* (Academic, New York, 1985).
2. Rakic, A., Djurisic, A., Elazar, J. & Majewski, M. Optical properties of metallic films for vertical-cavity optoelectronic devices. *Applied Optics* **37**, 5271–5283 (1998).
3. Sweatlock, L. A. *Plasmonics: Numerical Methods and Device Applications*. PhD thesis, California Institute of Technology, Pasadena (2008).
